# Updated Principles of Surgical Management of Pancreatic Neuroendocrine Tumours (pNETs): What Every Surgeon Needs to Know

**DOI:** 10.3390/cancers13235969

**Published:** 2021-11-27

**Authors:** Charles de Ponthaud, Fabrice Menegaux, Sébastien Gaujoux

**Affiliations:** 1 Department of General, Visceral, and Endocrine Surgery, Pitié-Salpêtrière Hospital, AP-HP, Bat. Husson Mourier, 47-83 Boulevard de l’Hôpital, 75013 Paris, France; charles.de-ponthaud@aphp.fr (C.d.P.); fabrice.menegaux@aphp.fr (F.M.); 2 Department of Hepato-Biliary and Pancreatic Surgery and Liver Transplantation, AP-HP, Bat. Husson Mourier, 47-83 Boulevard de l’Hôpital, 75013 Paris, France; 3 Paris-Sorbonne University, 21 rue de l’Ecole de Médecine, 75006 Paris, France

**Keywords:** pancreatic neuroendocrine tumours, parenchyma sparing pancreatectomy, minimally invasive approach, lymphadenectomy

## Abstract

**Simple Summary:**

In this narrative review, we update the surgical management of pancreatic neuroendocrine tumours (pNETs) and highlight key elements in view of the recent literature. These tumours are rare and suffer from a lack of data and randomized controlled trials. The pNETs management is difficult due to their heterogeneity and the risks associated with pancreatic surgery. Innovative managements such as “watch and wait” strategies, parenchymal sparing surgery and minimally invasive approach are emerging. The correct use of all these therapeutic options requires a good selection of patients but also a constant update of knowledge.

**Abstract:**

Pancreatic neuroendocrine tumours (pNETs) represent 1 to 2% of all pancreatic neoplasm with an increasing incidence. They have a varied clinical, biological and radiological presentation, depending on whether they are sporadic or genetic in origin, whether they are functional or non-functional, and whether there is a single or multiple lesions. These pNETs are often diagnosed at an advanced stage with locoregional lymph nodes invasion or distant metastases. In most cases, the gold standard curative treatment is surgical resection of the pancreatic tumour, but the postoperative complications and functional consequences are not negligible. Thus, these patients should be managed in specialised high-volume centres with multidisciplinary discussion involving surgeons, oncologists, radiologists and pathologists. Innovative managements such as “watch and wait” strategies, parenchymal sparing surgery and minimally invasive approach are emerging. The correct use of all these therapeutic options requires a good selection of patients but also a constant update of knowledge. The aim of this work is to update the surgical management of pNETs and to highlight key elements in view of the recent literature.

## 1. Introduction

Pancreatic neuroendocrine tumours (pNETs) are a group of rare neoplasms with an incidence of 1–2/100,000 inhabitants/year. They represent 2% of all pancreatic neoplasms. Their incidence is however increasing with more and more localized forms or incidentaloma at the time of the diagnosis, explained by the improvement of diagnostic cross-sectional imaging [1].

**Figure 1:**
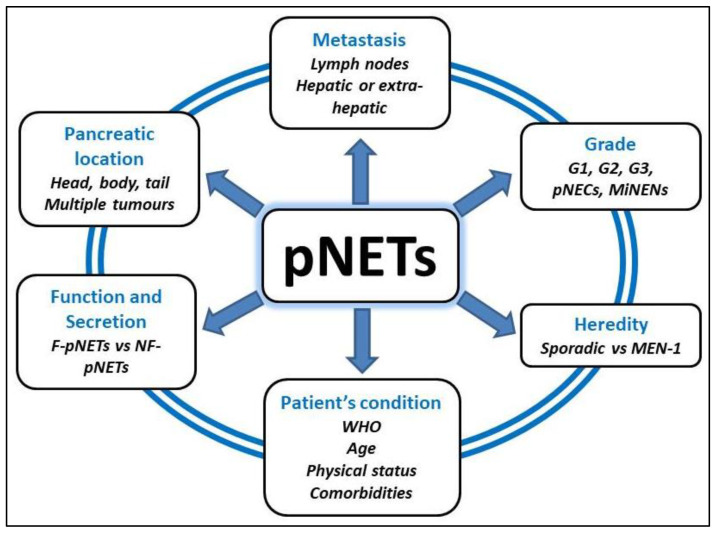
Different factors that have to be considered in the surgical management of patients with pNETs.

The management of pNETs is particularly complex, given their great heterogeneity. They therefore require the assistance of an expert centre to take into account all these factors in the therapeutic decision (Figure 1). Thus, pNETs are divided into two categories depending on clinical presentation due to hypersecretion of hormones [2]: the non-functional pNETs (NF-pNETs) that represent 60–90% of pNETs are frequently diagnosed as incidentaloma (82%) [3] vs. the functional pNETs (F-pNETs) (Table 1). The pNETs can also be integrated in a genetic syndrome or be sporadic and are stratified in different grades, according to biological and histological characteristics (Table 2). Lastly, pNETs can be diagnosed as localized, locally advanced or metastatic (50% of cases) with sometimes multiple tumours in the same patient. Whenever possible, pancreatic resection remains the only curative option for patients with pNETs. Nevertheless, this therapeutic decision should be chosen carefully as pancreatic surgery is a technically difficult surgery for selected patients with a significant rate of postoperative complications. This review aims to describe the main principles of surgical management of patients with pNETs, incorporating recent literature and highlighting some key points and grey areas.

## 2. Surgical Indications

Surgery is indicated in patients with pNETs to prevent malignant transformation or metastases and to reduce symptoms due to hormone hypersecretion or local invasion (biliary and pancreatic duct stenosis, portal thrombosis, duodenal stenosis, etc.). The decision has to systematically be discussed in a multidisciplinary network of specialised surgeons, gastroenterologists, oncologists and radiologists.

### 2.1. When Surgery Might Not Be an Option?

According to the literature, there are two situations in which pancreatic surgery is not systematic and may even be deleterious. The first case concerns the small (≤2 cm), sporadic and asymptomatic G1 NF-pNETs, for which a simple surveillance (“watch and wait” strategy) is an option validated by the European Neuroendocrine Tumour Society (ENETS), the US National Comprehensive Cancer Network (NCCN), and the gastro-entero-pancreatic neuroendocrine neoplasms ESMO [5,6,7,8,9,10,11]. This decision is based in particular on the benefit-risk balance of surgery. In fact, in most surgical series, there is still a non-negligible risk of lymph node involvement for these tumours, about 10% [12], but which should be compared with the significant morbidity, mortality and functional consequences of pancreatic surgery [13]. This strategy is conditioned by several criteria:

- Typical radiological features of low-grade pNET: marked arterial phase contrast on CT-scan or MRI, positive somatostatin receptor imaging, negative PET-FDG.
- Histological proof of pNET well-differentiated G1, eventually expandable to G2 but with Ki67 < 5%.
- No suspicion of lymph node or distant metastasis.
- No pancreatic or biliary ductal dilatation on imaging.
- No signs of radiological progression on follow-up.

The second situation in which pancreatic surgery seems inadvisable is matters of clinical common sense but is nevertheless worth recalling. We mention in particular: the presence of extra-hepatic metastases, massive hepatic invasion, presence of a cavernoma, frail patients with a high risk of post-operative complications, pNET G3 well differentiated and pancreatic neuroendocrine carcinomas (pNECs) (Table 2). Nevertheless, in the case of a locally advanced pNET of the head of the pancreas, invasion of the superior mesenteric vein or the portal trunk by the tumour does not systematically contraindicate pancreaticoduodenectomy, whose results in terms of overall survival with or without venous resection are identical [14].

### 2.2. When Surgery Is Required

Finally, once we have eliminated the situations where surgery could be avoided, all other cases are formal indications for surgical treatment.

#### 2.2.1. NF-pNETs

A surgical indication is established for NF-pNETs larger than >2 cm, symptomatic and/or associated with biliary or pancreatic ductal dilatation. In these situations, the risk of metastases becomes significant and justifies pancreatectomy [5,15].

#### 2.2.2. F-pNETs

For patients presenting an insulinoma, a pancreatic resection is always indicated [5,7]. On the one hand, this is a medical emergency because severe and iterative episodes of hypoglycaemia can be life-threatening or can greatly deteriorate the quality of life. On the other hand, the majority of insulinomas are sporadic and benign in over 90% of cases. Malignant insulinomas represent less than 10% of cases [16] and therefore eligible for parenchymal sparing surgery (see below). Besides, resection of insulinoma has a high rate of cure (almost 100% of cases) [17]. Ablation therapies by endoscopic ultrasound have been proposed as a possible option to surgery for patients in whom the surgical risk would be too great [18,19].

Surgery is also indicated for sporadic gastrinoma due to the risk of malignant lesion of 60–90% [20]. After surgery, disease free survival rate is 35% at five years, with an overall survival at 10 years of 94% [21]. For others F-pNETs, resection is indicated due to the non-negligible risk of malignancy for glucagonoma (50–80%), VIPoma (60–80%) and somatostatinoma (70–92%) [4].

## 3. Where Patients with pNET Should Be Operated?

The relationship between the volume of activities of a centre and survival after pancreatic surgery has been evaluated in several studies [22,23]. In addition, all the consensus guidelines reaffirmed the need to manage these patients with pNETs in expert centres with surgeries performed by surgeons and teams trained and experienced in this kind of pathology [4,5,24,25].

## 4. Medical Preparation before Surgery and the Role of the Anaesthetist

Before realizing any kind of surgery in patients with pNET, it is important to correctly characterise the lesion and its hormonal secretion in order to set up an adapted therapy and avoid possible complications caused by the stress of the surgery [24,26]. In this section, we do not pretend to carry out a review of the literature on the complete anaesthetic management of these patients, but simply to highlight certain elements whose management is common between surgeon and anaesthetist.

### 4.1. Gastrinoma

Due to the hypersecretion of gastrin [4,5], these patients are particularly at risk of digestive perforation and bleeding. The acid hypersecretion persists even after removal of the tumour by residual hypertrophy of gastric mucosa. In fact, during and several months after surgery, patients need high doses of PPIs to efficiently control it.

### 4.2. Insulinoma

Perioperative blood glucose control is important because of the risk of hypoglycaemia caused by preoperative fasting or hyperglycaemia following insulinoma resection. In addition, in order to ensure complete resection of insulin-secreting tissue, or to ensure that another tumour location of the insulinoma is not overlooked, some authors have proposed intraoperative monitoring of insulin level using a rapid insulin immunochemiluminescent assay [27]. Before surgery, preoperative diazoxide is sometimes used to control hypoglycaemia in 50–60% of cases, but can cause many side effects (water retention, hirsutism, renal malfunction, etc) [28]. In contrast to others pNET, SSAs efficiency is unclear with around 50% of effectiveness. Moreover, it could lead to more severe hypoglycaemias by suppression of the contra-regulatory hormones [29]. Patients with insulinoma require intravenous glucose 10% infusion during their preoperative fasting.

### 4.3. Glucagonoma

Patients have a major risk of deep vein thrombosis up to 30–50% and should preoperatively receive prophylactic anticoagulation treatment [30]. The anaesthetist and the surgeon should also keep in mind other events that may compromise the successful completion of the surgery: diabetes, chronic diarrhoea with dehydration (15%), dilated cardiomyopathy, anaemia (49%) and neuropsychiatric symptoms, which are often overlooked [31].

### 4.4. VIPoma

Patients have massive diarrhoea responsible for hydroelectrolytic disorders and dehydration [30]. Ionic and hydric supplementation is essential with SSAs before underwent the surgery [4].

**Take Home Messages**

✓ Surgery is the backbone for the curative treatment of localized pNET G1 and G2
✓ A “watch and wait” strategy is possible for NF-pNETs ≤ 2 cm and asymptomatic.
✓ Surgery is recommended for all F-pNETs and for NF-pNETs > 2 cm
✓ Surgery has to be realized in expert centres and by surgeons trained and experienced after discussion during a multidisciplinary network.
✓ For F-NETs, a medical treatment before surgery is required. This will include: correcting fluid and electrolyte disorders and dehydration, balancing blood glucose.

## 5. Intraoperative Strategy

### 5.1. Standard Pancreatectomy (SP) vs. Parenchyma Sparing Pancreatectomy (PSP)

SP is a non-conservative surgery for pancreatic tumours. It includes pancreaticoduodenectomy (PD) for tumours located at the head of the pancreas, and the distal pancreatectomy (DP) for tumours usually on the left side of gastro-duodenal artery. Their advantages lie in the possibility of resecting the tumour with sufficient lateral margins, associated with formal lymphadenectomy. However, these are major surgeries, with a non-negligible risk of complications and which lead to an extensive resection of the pancreas, source of postoperative endocrine and exocrine pancreatic insufficiencies [32,33].

PSP is a conservative surgical treatment which includes the central pancreatectomy (CP) for tumours located on the isthmus, and enucleation that is indicated for superficial tumours far enough from the main pancreatic duct (2–3 mm of distance from it). From an oncological point of view, PSP only allows a limited resection in terms of margin around the tumour, without the possibility of performing a satisfactory lymph node removal. However, this surgery also presents a significant risk of postoperative complications, with a rate of postoperative pancreatic fistula (POPF) sometimes even higher than SP. Nevertheless, due to the non-extensive resection, endocrine and exocrine pancreatic functions are preserved [34]. This is particularly important as a recent retrospective study found an association between postoperative diabetes mellitus exacerbation and increased risk of recurrence of pNET after pancreatectomy, in multivariable analysis (HR = 2.35) [35] (Table 3).

In general terms, when a surgery is indicated, SP is feasible in all situations. Nevertheless, PSP has to be preferred whenever it possible, in order to decrease endocrine and exocrine pancreatic insufficiencies, taking into account the surgeon’s experience.

Thus, the choice between these two categories of surgery will be based on three axes:

- The oncological objectives in terms of margins and especially lymph node dissection.
- The tumour location.
- The patient’s condition: age, comorbidities, curative anticoagulation, risk of pancreatic fistula, interest in preserving pancreatic function, etc.

#### 5.1.1. NF-pNETs

Due to the good prognosis of small (<2 cm) asymptomatic NF-pNETs, if a “watch and wait” strategy is not chosen, PSP seems to be a good alternative with satisfactory oncological results [34,44,45]. In a multi-institutional retrospective review which compared enucleation and SP in 122 patients with pNET ≤ 3 cm and without nodal or metastatic disease, 5-year survival rate was similar [46]. However, since the risk of lymph node invasion in these tumours is 10%, lymph node picking seems a reasonable procedure. In all other cases, a SP remains recommended.

#### 5.1.2. Insulinoma

Sporadic insulinoma is a good prognosis tumour, often solitary with a small size [9,17,47]. PSP (notably enucleation) is an excellent indication, provided that the tumour has been correctly located, if needed with the use of intraoperative ultrasound [48].

Malignant insulinoma are rare and frequently associated with larger and atypical tumours. Its treatment requires an extensive lymph nodes removal and a large parenchyma resection to obtain a R0 resection. A PSP is therefore not advisable in this case and a SP should be carried out.

#### 5.1.3. Gastrinoma

Gastrinomas may be unique or multiple and are often (more than 70% of cases) located in the duodenum. In fact, one of the most important elements in this disease is to locate the lesion(s). After extensive preoperative exploration, several methods are possible intraoperatively to localize associated lesions: duodenoscopy with transillumination and bi-digital palpation with duodenotomy for duodenal locations and ultrasound for pancreatic locations [49]. A study in 2004 demonstrates that routine use of duodenotomy in sporadic gastrinoma improved the rate of gastrinoma diagnosis from 76% to 98%, with an increase of short and long-term cure rate [50].

Surgical resection associated with lymph nodes removal has been shown to increase overall survival in patients with sporadic gastrinoma [5,20,21]. Preferential location of gastrinoma is in an area so-called “Stabile and Passaro” triangle [51]. This area is bounded by the cystic duct on the top, the junction of the second and third duodenum on the right and the isthmus of the pancreas on the left. Thus, surgery could be either a PD or resection of the primary tumour by duodenectomy associated with a lymphadenectomy. In the past, if the gastrinoma was not found, a parietal cell vagotomy or total gastrectomy was performed. With the advent of PPIs, these procedures are no longer needed.

Furthermore, patients with Zollinger-Ellison Syndrome (ZES) and a negative imaging should have surgical exploration by an experienced surgeon [5]. Indeed, a 2012 cohort study [52] reported that in these patients, gastrinoma was found intraoperatively in 98%, 46% were cured and the overall 20-year survival after resection was 71%. Conversely, as discussed in a previous paragraph, this rule only applies to sporadic gastrinomas as well as to gastrinomas in the context of MEN-1 but that do not meet the surveillance criteria as we will see in the paragraph on the MEN-1.

#### 5.1.4. Others F-pNETs

Concerning VIPoma, somatostatinomas and glucagonomas, all patients with localized tumours on preoperative imaging should undergo surgical exploration. However, these lesions present synchronous metastases more than half of cases at the diagnostic and the surgical management will depend on their resectability [53,54].

### 5.2. Surgical Approach: Minimally Invasive or Open Pancreatectomy?

Today, minimally invasive surgery has become a standard of care for many procedures, reducing postoperative pain, length of stay, blood loss and complications without compromising oncological outcomes [55,56,57]. However, its diffusion in pancreatic surgery is slower due to the complexity and morbidity of the procedures. Randomized controlled trials are finished or underway to evaluate the minimally invasive approach in pancreatic surgery (Table 4). For now, there is no consensus concerning the indications of the minimally invasive approach for pancreatic resection in pNET [58].

Laparoscopic approach in PD presents several inconveniences compared with open approach, including a long learning curve and a limited range of motion with poor surgeon ergonomics specially to perform anastomosis. Besides, no superiority or equivalence of the laparoscopic approach to the Whipple procedure has been demonstrated [59]. LEOPARD-2, a multicentre randomised controlled trial comparing laparoscopic vs. open approach, was stopped prematurely due to an excess of mortality in the laparoscopic group (10% against 2%) [60]. The same results have been found in a practice evaluation among 7061 patients with a mortality rate of 5.1% vs. 3.1% in open approach [61]. Robotic surgery has several benefits obtained by three-dimensional vision and rotation in all planes of the instruments, compared with laparoscopic approach. These applications would be particularly interesting in PD with reconstruction and anastomosis times [62,63]. Nevertheless, laparoscopic or robotic PD could not be recommended at the present point.

For DP, the laparoscopic approach is now widely practised. The literature, including two randomized controlled trials and their meta-analysis [64,65,66] suggests better short-term results for laparoscopy with fewer severe complications, fewer DGE, shorter length of stay, faster functional recovery, without additional costs. Long-term results, particularly in terms of oncology, are still awaited in these studies. The robot-assisted approach in DP seems less attractive due to the absence of reconstruction and the cost of robotic approach.

For enucleation and central pancreatectomy, laparoscopy would appear from retrospective studies to have at least equivalent results to laparotomy [67,68].

Thus, the minimal invasive approach has been progressively proposed for DP and PSP for pNETs. Various studies showed a benefice on short-term postoperative outcomes of a minimally invasive approach in the NF-pNETs and insulinoma without compromising oncologic results [64,69,70]. A meta-analysis on 907 patients demonstrated a shorter length of hospital stay for laparoscopy group compared with open approach in pancreatectomy for pNETs, without difference concerning others postoperative outcomes [71]. The robotic or laparoscopic approach seems to have the same postoperative results on DP for pNETs with perhaps less blood loss and more splenic preservation in the robot approach [40,72,73,74].

The role of laparoscopy in gastrinoma surgery is controversial due to the necessity to explore the abdominal cavity to search multiple lesions which were not localized preoperatively, and notably the duodenum [75].

**Table 4:** Randomised-controlled trials (RCTs) which evaluate minimally invasive approach in pancreatic surgery (not specific to pNET).

RCTs	Distal Pancreatectomy	Pancreaticoduodenectomy
Laparoscopy	LAPOP trial [64] LEOPARD trial [40] DIPLOMA trial [72]	LEOPARD-2 trial [60] PLOT trial [57] PADULAP trial [76] F. Nickel, 2020 [59] TJDBPS01 [77]
Robot-assisted	None	None

### 5.3. The Practical Implications of a Biliary-Digestive Anastomosis in the Therapeutic Arsenal

NETs associated with liver metastasis are a major challenge. Surgery is the treatment of choice for resectable liver metastasis from well-differentiated NET with a 5-year survival rate of 60–80% [78,79]. However, for patients with unresectable liver metastasis, other therapies exist, led by liver transarterial embolization [80]. The latter includes selective internal radiation therapy and transarterial chemoembolization.

Unfortunately, these techniques (excepted selective internal radiation therapy) are not recommended for patients with a biliary-digestive anastomosis (e.g., in PD), due to the high risk of sepsis and biliary ischaemia [80,81]. Indeed, a retrospective study of 397 transarterial chemoembolization showed that the risk of developing liver abscesses was increased with an odds ratio of 894, in patients with a biliary-digestive anastomosis [82].

Thus, this consideration should be taken into account in patients undergoing PD for pNET where a biliary-digestive anastomosis will inevitably be performed. This will probably narrow of the scope of subsequent therapies in the case of hepatic metastatic disease.

### 5.4. Cholecystectomy and NETs

SSAs are often the first line treatment for neuroendocrine disease. Unfortunately, several adverse events have been reported, mainly: gastrointestinal disturbances with pancreatic enzyme insufficiency (diarrhoea, abdominal pain, nausea or vomiting), vitamin B12 deficiency, hyperglycaemia in 5%, and biliary gallstones [83]. Besides, in a large retrospective analysis of 478 patients treated with SSAs, biliary stone disease was observed in 27% of cases [84]. Biliary stones have been also reported in 10% of patients with lanreotide and 14% with octreotide in the CLARINET and PROMID trials, respectively [85,86]. In addition, several cases of ischaemic cholecystitis have been reported in the literature following chemoembolization [80]. Thus, prophylactic cholecystectomy during surgery for NET may be considered in this population at high risk of developing biliary pathology [87].

### 5.5. Lymph Nodes Removal and NETs

Pancreatic NETs are frequently diagnosed with regional lymph nodes metastases. Moreover, the presence of positive lymph nodes and their number have important prognostic value and predictors of recurrence after pancreatectomy [88,89,90,91]. There is a consensus that if standard pancreatic resection is indicated, it should be associated with standardised lymph node dissection with a minimum number of 12–13 nodes resected [92,93]. Para-aortic lymph nodes removal in pNETs surgery does not seem to be recommended.

**Take Home Messages**

✓ SP with lymph nodes removal is the referral surgery. However, PSP without lymph nodes removal could be possible in small NF-pNET ≤ 2 cm and small insulinoma (enucleation).
✓ The surgeon’s decision should take into account the characteristics of each of the surgical procedures and the main complications, including the risk of POPF and exocrine and endocrine pancreatic insufficiencies.
✓ The minimal invasive approach has to be preferred as soon as possible, except for PD. The place of the robot-assisted surgery has yet to be evaluated.
✓ It is important to routinely look for multiple lesions during gastrinoma surgery, including notably the duodenum.
✓ Cholecystectomy may be considered during surgery for NETs to prevent biliary gallstones caused by SSAs and ischaemic cholecystitis after chemoembolization.
✓ Chemoembolization is not recommended in patients with biliary-digestive anastomosis due to the high risk of sepsis and biliary ischaemia.

## 6. Place of Surgery in Metastatic Disease?

Metastatic disease is common in pNETs. Indeed, approximately 50% of patients have metastatic disease at the time of diagnosis [78]. Metastases are predominantly found in the liver (LM) [94] and lymph node. Extrahepatic metastatic disease, notably bone metastases that occur in 12% of cases [95], is a contraindication to surgery and has a poor prognosis [78]. In selected patients with synchronous bilobar LM, surgical resection can be performed in two stages to achieve complete resection of the primary tumour and LM with satisfactory long-term survival [96]. Of course, this extensive procedure is conditioned by the resectability of the LM and must be balanced against the almost inevitable risk of long-term metastatic recurrence.

### 6.1. Resection of the Primary pNET in Patients with Liver Metastases

Despite the lack of prospective randomised controlled data in the literature to answer this question, several retrospective studies showed an improvement of survival rate after synchronous resection of primary pNET and resectable LM [79,97,98,99].

In the setting of unresectable LM, there is no clear consensus on the place of resection of the primary pNET, even if it is avoided by most teams nowadays. The surgical indication, although rare, can be discussed in order to prevent possible local complications (portal hypertension, obstructive complications, hormonal refractory symptoms, etc.) or to anticipate a future liver transplantation. The most appropriate candidates may be those with a low-grade, very stable pNET located outside the head of the pancreas in order to avoid morbid surgery (PD) that would contraindicate subsequent locoregional liver treatments. Some retrospective studies suggest that resection of primary pNET in cases of unresectable metastases may be associated with a survival benefit [100,101]. A meta-analysis has been realized in 885 patients with pNET and unresectable LM. Among them, 252 underwent a surgical resection with a reduction of 5-year mortality rate (OR = 0.38) [101]. Lastly, an improvement in overall survival was also observed in patients who had destruction of LM by local ablative techniques such as thermal hepatic ablation, radiofrequency ablation or microwave ablation even if this remains also highly controversial [102].

### 6.2. Liver Transplantation

Liver transplantation (LT) is not presently a standard for unresectable neuroendocrine metastases of the liver (<1% of NETs with metastases) and remains controversial.

Despite the paucity of data in the literature and the absence of a randomised controlled trial, several series can be mentioned [103,104,105,106]. An important retrospective multicentric international study [107] between 1982 and 2009 evaluated results of 213 patients who underwent a LT for NETs. Mortality rate was 10%, overall survival and disease-free survival at 5 years were respectively 52% and 30%, with a survival rate for patient with primary pNETs that was worse than patients with primary gastrointestinal NETs (44% vs. 62%). Finally, 60% of patients had disease recurrence. In a report from the United Network for Organ Sharing database [108], 150 LT were included between 1988 and 2008. In this series, the 5-year overall survival rate was 49%, with no statistical difference found compared to the 4693 LT for hepatocellular carcinoma during the same period (*p* = 0.20).

Thus, the place of LT for NETs remains poorly defined, especially in the setting of new systemic treatment, and poorly evaluated, notably in the context of organ shortage. But, there appears to be an improvement in survival rate after LT in highly selected patients [79,106]. Therefore, it has been developed patient selection criteria, without a clear consensus, [103,109,110], including:

- Age < 55 years
- Primary tumour drained by the portal system (pancreas and intermediate gut) already removed with a curative resection before LT
- <50% involvement of liver parenchyma
- Stable disease for at least 6 months before LT
- Ki 67 < 10%
- Low-grade and well differentiated tumour (G1, G2)
- Absence of extrahepatic disease
- No extra-hepatic combined resection.

However, recent high-quality data are needed to confirm the data and to determine the best timing and patient selection of LT compared to other therapeutic options.

### 6.3. Surgery in Unresectable pNETs

Peritoneal carcinomatosis (PC) affects 6–30% of gastro-entero-pancreatic NENs. Tumour reduction surgery may be proposed in selected patients. Some studies have shown an interest in performing a cytoreduction of peritoneal carcinomatosis in terms of survival when >90% of the disease could be resected [111,112,113]. In a retrospective study based on a National Cancer Data, the 5-year survival rate for patients with a metastatic disease who underwent surgical debulking, was 86% [114]. However, these are mainly studies of patients with small bowel NETs. Lastly, there is no evidence for the value of hyperthermic intraperitoneal chemotherapy in this indication [111]. Once again, this extensive resection has to be balanced against the almost inevitable risk of long-term metastatic recurrenc due to persistent microscopic tumours in all patients [115].

**Take Home Messages**

✓ The first site of distant metastases is the liver, whose resectability will determine management.
✓ In the case of resectable LM combined surgery to remove the primary tumour and all hepatic metastases is recommended.
✓ Primary tumour resection in the presence of unresectable LM is not consensual but may be beneficial in highly selected cases.
✓ Liver transplantation is a surgical alternative with very strict criteria.

## 7. What about Multiple Endocrine Neoplasia Type 1 (MEN-1)?

MEN-1 is an autosomal dominant hereditary syndrome with a prevalence of 2/100,000 inhabitants, caused by a mutation in the *MEN-1* tumour suppressor gene on the long arm of chromosome 11 (11q13) [116,117]. The most common MEN-1 lesions (with their penetrance in parentheses) are: primary hyperparathyroidism (90%), Entero-pancreatic tumour (30–70%) with gastrinoma (40%), insulinoma (10%), NF-pNETs (20–55%), and others F-NETs (2%), anterior pituitary tumour adenoma (30–40%), adrenal cortical tumour (40%), thymic (2%) and bronchial carcinoid (2%), gastric enterochromaffin-like tumour (10%) [116,118]. The pNETs represent one of the principal causes of death in patients with MEN-1 [119]. Furthermore, the probability of diagnosing MEN-1 in patients with gastrinoma, insulinoma, VIPoma, glucagonoma and somatostatinoma are respectively 20%, 5%, 6%, 1–20% and 45% [3,4].

Despite various published guidelines [4,120,121], the management of these tumours is difficult to standardize. Indeed, most if not all of patients with MEN-1 will have pNETs, knowing that in the vast majority of cases (not to say 100%) there are multiple with F-pNET and NF-pNET possibly co-existing in the same patient. [119].

### 7.1. Gastrinoma

Approximately 40% of patients with MEN-1 syndrome have a gastrinoma whose primary location is duodenal. These are typically multifocal and small. Gastrinomas are associated with lymph node involvement in 40–60% of cases. Besides, other rarer intra-abdominal locations are described in the literature: pancreatic (rare and usually unique), extra-hepatic biliary tract and liver, stomach, mesentery, renal capsule, splenic hilum and omentum [122].

Once again, a “wait and see” strategy is recommended for small gastrinoma (≤2 cm) in MEN-1 with no metastasis [123,124]. This option is all the more legitimate as typical gastrinoma in MEN-1 patients is preferentially multiple with an excellent survival rate of up to 90–100% at 15 years [5,125]. Gastric hypersecretion can be correctly controlled medically by PPI treatment [126]. However, the question arises as to the adverse effects induced by the long term use of high dose PPI [127], such as: enteric infections, microscopic colitis, chronic atrophic gastritis, malabsorption, kidney disease, etc. Histamine-2 receptor antagonists (H2Ra) are an alternative to PPIs but with less clinical efficacy. Nevertheless, it would appear that H2Ra are associated with fewer long-term side effects than PPIs, although there is some contradiction in the literature [128,129]. Concerning the place of SSA in the management of gastrinomas, here again, they suffer from the rarity of their prevalence with little data in the literature, preventing us from defining a consensus. Several studies have already demonstrated the value of SSA in the management of NETs in terms of tumour control and disease free survival (CLARINET and PROMID). Although few gastrinomas have been included in these studies, we know the importance of somatostatin receptor expression in this tumour, which allows us to easily extrapolate the results of SSAs to gastrinomas. Moreover, Ramundo et al. confirmed the impact of SSA on 20 patients with early-stage MEN-1 related duodeno-pancreatic NETs, with a tumour response in 10% and a prolonged clinical and biochemical response in all patients [130]. Finally, several case series in the literature have demonstrated the benefit of SSA on gastrin level and symptoms of ZES. Nevertheless, PPIs remain the standard treatment due to their widely proven and long-lasting efficacy, as well as their oral availability [131].

Surgery is indicated in the other cases and if there is a nodal involvement [132] due to the fact that 25% of gastrinoma tumours finally show an aggressive behaviour. Given that the preferential location of gastrinoma in MEN-1 is the duodenum, its exploration (by endoscopic transillumination or duodenotomy) should be performed intraoperatively. Management of MEN-1-associated gastrinoma higher than >2 cm is controversial most notably because of its duodenal location. Indeed, there is no clear consensus on the choice between limited resection with duodenotomy ± lymph node removal ± DP vs. PD. Theoretically, the first option allows resection of the tumour and prevention of metastatic progression, with less morbid surgery but without achieving long-term cure. The second option, i.e., PD, avoids the risk of duodenal recurrence, but at the expense of more morbid surgery, while the risk of developing F-pNETs on the remaining pancreas is still present. However, the reality is more complex. In a limited cohort of 22 patients with duodenal gastrinoma associated with MEN-1, even though disease–free survival rate was higher after PD than limited resection, there was no difference on overall survival [133]. Besides, in literature, biochemical cure rate, defined by negative secretin test, is higher after PD (between 77% and 100% in literature) than after limited resection (nearly 30%). On the opposite, several authors support the excellent functional and oncological results following surgery with limited resection and complete duodenal exploration [134,135,136]. In the future, better tumour staging and better prediction of the aggressiveness of gastrinomas in MEN-1 will be key elements in the choice of intervention.

### 7.2. NF-pNETs

The prevalence of NF-pNETs in patients with MEN-1 is between 30–75%. Their management is similar to the sporadic NF- pNETs [123,124].

### 7.3. Insulinoma

Approximately 10% of patients with MEN-1 have an insulinoma whereas 5% of patients with insulinoma have a MEN-1. Unlike sporadic insulinoma which are mostly isolated therefore eligible for enucleation, insulinomas in the context of MEN-1 are frequently multiple. Thus, two surgical approaches are possible: either multiple enucleations of the main tumours after a thorough search for all pancreatic tumour locations, or an enlarged DP associated with enucleation of possible lesions of the head of the pancreas. Besides, enucleation is associated with a higher rate of hypoglycaemia recurrence compared to DP, respectively 33% versus 8.7% [137,138]. The difficulty remains to preoperatively identify which ones of the multiple pNETs are secreting insulin.

**Take Home Messages**

✓ MEN-1 is an autosomal dominant hereditary syndrome. Several lesions are part of this syndrome: primary hyperparathyroidism, pNETs, anterior pituitary tumour adenoma, adrenal cortical tumour, thymic and bronchial carcinoid.
✓ Multifocal pNETs are always present in the same patient, with different types of tumours.
✓ A “wait and see” strategy is recommended for small gastrinoma (≤2 cm) in MEN-1 with no metastasis, and gastric hypersecretion can be controlled medically by PPI treatment.
✓ PD is probably the best option for patients with Zollinger–Ellison syndrome (ZES) in MEN-1 context because gastrinomas tend to be numerous and located in the duodenum.

## 8. Surveillance

### 8.1. Survival Rate and Recurrence

Recurrence rate for pNETs resected is 12–25% at 5 years and up to 70% at 15 years [139,140,141,142]. Some authors have proposed scores to predict recurrence within 5 years after curative resection for patients with a G1 or G2 NF-pNET based on main recurrence risk factors, notably: positive lymph nodes, perineural invasion and tumour G2 [140,143]. Other nomograms have also been proposed to predict the risk of recurrence in patients who have had liver resection for LM from pNETs [144].

For insulinoma, the risk of recurrence in patient with MEN-1 was 21% at 10 years against 5% at 10 years in patient with sporadic insulinoma [145]. Concerning patients with gastrinoma, 10-year survival rate was 30% with LM and 83% without LM [146]. The level of fasting serum gastrin (FSG) at the time of diagnosis may be useful in planning the extent of sporadic gastrinoma and to estimate prognosis [147]. For somatostatinoma, the 5-year postoperative survival rate is about 75%, but there is few data in the literature on this. The same applies to VIPoma and Glucagonoma.

### 8.2. Follow-up of pNETs after Resection

Follow-up for patients who underwent a resection of pNETs, requires a careful monitoring with clinical exam, biological markers and radiological exam (CT-scan or MRI), every 6 months for pNETs G1 or G2 with Ki67 < 5%, and every 3 month for pNETs G2 with Ki67 > 5% [11]. This monitoring should continue over time, usually at least 10 years after pancreatectomy. Somatostatin receptor imaging should be repeated every 2 years if positive or earlier if progression is suspected [5].

Surveillance interval might be adapted to the tumour aggressiveness with the aim of personalized medicine, reduction of extra costs and radiological irradiation. Indeed, a study of 1006 patients defined a recurrence risk score (RRS) for resected well/moderately differentiated NF-pNETs, based on the 4 risk factors predictive of recurrence found in multivariate analysis (symptomatic tumour, size > 2 cm, Ki67 > 20% and lymph nodes positivity). Patients were scored from 0–10 and divided into 3 groups according to the degree of risk. Based on this allocation, the authors propose an adjustment of oncological surveillance every 12-, 6-, 3-months for low-, moderate- and high-risk groups respectively [148].

Some authors have also proposed the use of postoperative blood measurement of neuroendocrine gene transcripts (NETest) for early assessment of surgical resection of pNETs [149]. But its value as a marker of recurrence remains to be demonstrated, unlike the chromogranin A assay, which is recommended by NANETS for post-operative follow-up [150].

## 9. Conclusions

The management of pNETs is complex and requires extensive medical and surgical coordination, which will be discussed at a dedicated multidisciplinary staff meeting in expert centres. The curative treatment of pNETs is largely based on surgery. The latter have to take into account many parameters: tumour characteristics, location, patient’s history, informed patient choice, etc. The consensus is that surgery should be performed routinely for NF-pNETs larger than 2 cm or for F-pNETs of any size. A “watch and wait” strategy can be proposed for asymptomatic NF-pNETs < 2 cm, as well as for gastrinomas less than 2 cm in the context of MEN-1. For pancreatic resection, the choice between a SP and a PSP is based on many elements, including tumour type, risk of lymph node invasion, patient’s history, subsequent use of chemoembolization, informed patient preference, etc. The laparoscopic minimally invasive approach is currently increasingly practised except for PD.

Further trials, including prospective randomised trials, are needed to refine the selection and surgical management of patients with advanced or metastatic disease.

## Figures and Tables

**Table 1:** Clinical characteristics of pNETs [3,4,5].

Neoplasms (Secretion)	Main Symptoms	Pancreatic Location
NF-pNETs (none)	Depending on local invasion: asymptomatic (87%), jaundice, pancreatitis, pain, bleeding, digestive obstruction	100%
Gastrinoma (Gastrin)	Zollinger-Ellison syndrome: Acid hypersecretion, peptic gastro-duodenal ulceration, diarrhea	25%
Insulinoma (Insulin)	Whipple’s triad (Symptomatic hypoglycaemia where restoration of normoglycaemia results in the disappearance of these symptoms), confusion, behavioral changes, visual troubles, coma	>99%
VIPoma (VIP)	WDHA syndrome (watery diarrhea, hypokalemia, achlorhydria)	90%
Glucagonoma (Glucagon)	Necrotic migratory erythema, hyperglycemia, dilated cardiomyopathy, anemia, neuropsychiatric symptoms	100%
Somatostatinoma(Somatostatin)	Diabetes mellitus, diarrhea, cholelithiases	55%

**Table 2:** World Health Organization (WHO) classification and grading of pancreatic neuroendocrine neoplasms (pNENs) 2017.

pNENs		Ki-67 Proliferation Index	Mitotic Index (/2mm^2^)
Well-differentiated Pancreatic neuroendocrine tumours (pNETs)	pNETs G1	<3%	<2
	pNETs G2	3–20%	2–20
	pNETs G3	>20%	>20
Poorly differentiated Pancreatic neuroendocrine carcinomas (pNECs)	pNECs (G3)	>20%	>20
	Small cell type		
	Large cell type		
Mixed neuroendocrine-non-neuroendocrine neoplasms (MiNENs)			

**Table 3:** Elements to differentiate surgical procedures.

	Standard Pancreatectomy (SP)		Parenchyma Sparing Pancreatectomy (PSP)	
	Pancreatico-Duodenectomy [36,37,38]	Distal Pancreatectomy [39,40]	Central Pancreatectomy [41,42]	Enucleation [43,44]
Lymph nodes removal	Yes	Yes (RAMPS)	±No *	No
Mortality	2–5%	<2%	0.4–1%	1%
Overall morbidity	40–50%	30–50%	40–70%	50–60%
CR-POPF (grade B + C)	10–20%	20–30%	25–35%	40%
DGE	17%	6–20%	2%	5–15%
New onset diabetes	16%	9–20%	4%	<7%
Exocrine pancreatic insufficiency	22–60%	10–30%	2%	<5%

CR-POPF: Clinically-relevant post-operative pancreatic fistula, grade B and C (ISGPS 2017). DGE: Delay Gastric Emptying; RAMPS: Radical antegrade modular pancreato-splenectomy. * A small lymph node dissection can be performed at least on the posterior side of the pancreas or if a resection of splenic vessels is done.

## Data Availability

This narrative review is based on previously published data.
